# Gut microbiome alterations in colitis rats after moxibustion at bilateral Tianshu acupoints

**DOI:** 10.1186/s12876-022-02115-1

**Published:** 2022-02-12

**Authors:** Qin Qi, Ya-Nan Liu, Si-Yi Lv, Huan-Gan Wu, Lin-Shuang Zhang, Zhan Cao, Hui-Rong Liu, Xiao-Mei Wang, Lu-Yi Wu

**Affiliations:** 1 Shanghai Research Institute of Acupuncture and Meridian, Shanghai University of Traditional Chinese Medicine, 650 South Wanping Road, Xuhui District, Shanghai, 200030 China; 2 Yueyang Clinical Medical College, Shanghai University of Traditional Chinese Medicine, Shanghai, 200437 China; 3 Zhejiang Institute for Food and Drug Control, Hangzhou, 310052 Zhejiang China; 4 Shanghai Institution of Gut Microbiota Research and Engineering Development, Shanghai Tenth People’s Hospital, Tongji University School of Medicine, Shanghai, 200072 China; 5 Shanghai Qigong Research Institute, Shanghai University of Traditional Chinese Medicine, Shanghai, 200030 China

**Keywords:** Ulcerative colitis, Moxibustion, Gut microbiota, Metagenomic

## Abstract

**Background:**

The pathogenesis of ulcerative colitis (UC) is closely related to the gut microbiota. Moxibustion has been used to improve the inflammation and gastrointestinal dysfunctions in gastrointestinal disorders such as UC. In this study, we investigated whether moxibustion could improve the gut microbial dysbiosis induced by dextran sulphate sodium.

**Methods:**

Twenty-five male rats were randomly assigned into five groups. The UC rat model was established by administering DSS solution. The rats in the moxibustion and normal rats with moxibustion groups were treated with moxibustion at Tianshu (bilateral, ST25) points, and the mesalazine group rats were treated with mesalazine once daily for 7 consecutive days. Disease activity index (DAI) and haematoxylin and eosin staining were used to evaluate the effect of moxibustion. Gut microbiota profiling was conducted by metagenomic high throughput sequencing technology. The gut microbiota composition, diversity and function were analyzed and compared using metagenomics methodologies.

**Results:**

The DAI scores and histopathology scores in the moxibustion and mesalazine groups were significantly decreased compared with the UC group (*P* < 0.01). Moxibustion treatment increased abundance levels of *Bacteroidetes*, *Actinobacteria*, *Ascomycota*, *Synergistetes* and decreased abundance of *Firmicutes*, *Proteobacteria*. At the genus level, the abundance of *Bacteroides*, *Bacteroides_bacterium_M7*, *Prevotella*, *Bacteroidales_bacterium_H2*, were increased and *Bacteroides_bacterium_H3*, *Parabacteroides*, *Porphyromonas*, *Alistipes*, *Parasutterella* were decreased in the UC group in comparsion with those in the NG group. Moxibustion increased the abundance of *Bacteroides* and *Bacteroides_bacterium_H3* and decreased *Bacteroides_bacterium_M7*, *Prevotella*, *Bacteroidales_bacterium_H2*. In UC group, the specie *Bacteroides_massiliensis* was negatively (*P* < 0.05) correlated with IL-23, *Bacteroides_eggerthii_CAG109* and *Bacteroides_eggerthii* were negatively (*P* < 0.05) correlated with TGF-β. And the species *Prevotella_sp_CAG1031* and *Bacteroides_bacterium_H2* were significant positively (*P* < 0.05) correlated with IL-23. In addition, compare with the normal group, genes involved in certain metabolic pathways, such as energy production and conversion, amino acid transport and metabolism, carbohydrate transport and metabolism, were under-represented in the UC group, and these changes in the metabolic pathways could be reversed by moxibustion treatment and mesalazine treatment.

**Conclusions:**

Our findings suggest that moxibustion treatment may protect the host from mucosal inflammation by modulating the intestinal microbiota community.

## Background

Ulcerative colitis (UC) is a chronic nonspecific intestinal inflammatory disease, which is clinical characterized by abdominal pain, diarrhea, rectal urgency and bleeding [1, 2]. The incidence of UC was steadily increasing with time around the world [3], and the patients generally have a reduced quality of life and are heavily burdened with medical costs [4]. However, the etiology and pathogenesis of the disease are not clear, and it is thought to be related to heredity, infection, immunity, environment and diet [5]. Evidence indicates that the intestinal microbiota is one of the key roles in the pathogenesis of UC [6–8].

Intestinal flora as the natural barrier of human body plays an important role in maintaining human health. Changes in internal and external environment can affect the structure of intestinal flora, if the intestinal environment is out of balance, such as *bifidobacterium* and *lactobacillus* significantly decreased, and *bacteroides* and *escherichia coli* increased in UC patients [9, 10], the intestinal bacteria and their metabolites continue to stimulate the host epithelial cells, affecting the intestinal mucosal immune system and inducing genetic predisposition in individuals with abnormal intestinal immune response, destructing of the structure and function of mucous membrane, eventually leading to the occurrence of UC.

Moxibustion, a common treatment in traditional Chinese medicine, is the burning of the herb moxa over acupuncture points. Modern experimental studies have shown that moxibustion has obvious advantage in adjusting immune function, which can correct abnormalities of cellular immune function, and improve, stabilize and coordinate the immune system [11, 12]. In the past 30 years, many scholars have conducted a lot of meaningful research on the mechanism of action of moxibustion in treating UC. It has been proved that moxibustion has a definite curative effect on UC [13–15], but its specific mechanism has not yet been fully understood. Our previous study found that *Bifidobacterium*, *Lactobacillus* were decreased and harmful bacteria *E. coli*, *Fusobacterium* were increased in the UC rats, and moxibustion can regulate the balance of those bacteria in UC rats [16]. To further understand the relationship between UC and intestinal flora, we used metagenomic high throughput sequencing technology to study the role of intestinal flora in the pathogenesis of UC and the role of moxibustion in the regulation of UC intestinal flora diversity.

## Methods

### Animals and experimental procedure

Twenty-five male Sprague–Dawley rats (weight, 180–220 g) were obtained from the Department of Laboratory Animal Science of Shanghai University of Traditional Chinese Medicine (No. SCXK (hu) 2012-0008). All animal experiments were carried out according to the Guidance Suggestions for the Care and Use of Laboratory Animals, formulated by the University Animal Care and Use Committee of Shanghai University of Traditional Chinese Medicine.

**Fig. 1:**
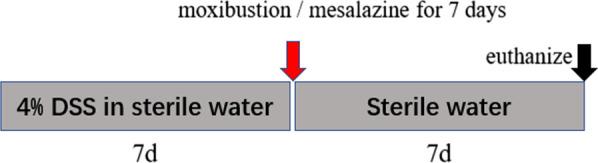
A schematic diagram of the experimental procedure

After one week of adaptive feeding, the rats were divided into five groups: Normal group (NG), UC model group (UC), moxibustion group (UC + MOX), mesalazine group (UC + MES), and normal rats with moxibustion group (NG + MOX), five rats were chosen in each group. UC model rats were established by 4% dextran sulphate sodium (DSS; Art.No.9011–18-1; MP Biomedical, CA, USA) solution for 7 days as described previously [17]. Moxibustion and mesalazine treatment will be started the next day after the completion of the model (Fig. 1). UC + MOX and NG + MOX: The Tianshu (bilateral, ST25) points were selected. The herb cakes were made of Chinese medicine powder and yellow wine into size of 0.6 cm in diameter and 0.45 cm in thickness. The moxa cone (diameter: 0.5 cm, high: 0.6 cm) was placed on the top of the herb cake, and the herb cake was placed on bilateral ST25 points, 2 moxa cones per time point for totally 7 days. UC + MES treated with mesalazine enteric coated tablet (Heilongjiang Tianhong Pharmaceutical Co., Ltd; Lot number: H20103359) dissolved in water by gavage at a daily dose of 300 mg/kg for 7 days. NG and UC groups only received the same fixation.

### Disease activity index (DAI)

The severity of colonic inflammation in rats was evaluated by monitoring manifestations daily, such as body weight, stool consistency and rectal bleeding. The DAI score for each animal was performed according to the follow parameters: the rate of weight loss ((0, no change; 1, 1–5%; 2, 6–10%; 3, 11–15%; 4, > 15%), viscosity of stool (0, normal; 2, loose; 4, diarrhea) and status of hematochezia (0, normal; 2, positive occult blood test; 4, visible bleeding) [18]. The DAI score = (rate of weight loss + viscosity of stool + status of hematochezia)/3.

### Sample collection and pathological observation

At the end of the treatment, all of the rats were anesthetized by intraperitoneal injection of 2% pentobarbital sodium, and then sacrificed by cervical dislocation. The fecal samples were collected and immediately stored in freezing tubes at -80 °C until DNA extraction. Colon tissues fixed in 4% paraformaldehyde fixative solution were dehydrated, embedded in paraffin, cut into 4 μm sections for haematoxylin and eosin (HE) staining and pathological observation. The histological score of the HE-stained colon specimens was performed according to the follow parameters: damage/necrosis, inflammatory cell infiltration, submucosal edema, and hemorrhage of mucosa. Colonic gross damage scores were recorded according to the severity of changes: 0, no change; 1, mild; 2, moderate; 3, severe [19]. The histological score was calculated as the sum of scores assigned for damage/necrosis, inflammatory cell infiltration, submucosal edema, and hemorrhage of mucosa.

### Bacterial genomic DNA extraction and sequencing

The Bacterial genomic DNA was extracted with QIAamp Fast DNA Stool Mini Kit (QIAGEN, Germany) from faecal samples as previously described [20]. DNA quality testing was assessed by 1% agarose gel electrophoresis. The metagenomic DNA library was prepared using PCR products according to the NEBNext Ultra DNA Library Prep Kit for Illumina (New England Biolabs). The PCR products were evaluated using an Agilent 2100 Bioanalyser (Agilent Technologies, Santa Clara, CA, USA) with an Agilent DNA 1000 kit according to the manufacturer’s recommendations. Amplicon sequencing was performed using Illumina HiSeq2500 (Illumina, San Diego, CA, USA).

### Quality control, alignment and assembly

Sequence contamination, such as the adaptor sequence, low quality bases and some impurity sequences, are often introduced during the sequencing. This data corruption must be removed to acquire the quality sequences for later analysis. The 3’ end of the read was trimmed and bases with average quality less than Q20 (the percentage of bases with a Phred value > 20) were removed. In addition, reads shorter than 60% of the original length were moved.

Then the clean reads were aligned with the NCBI database using SOAPaligner (version 2.21) by parameter of ‘–m 4 –r 2 –m 100 – × 1000’ for the detection of known bacteria, viruses, fungi and archaea [21]. The aligned reads were classified into six different levels including Kingdom, Phylum, Class, Order, Family, Species to count classification and abundance. The taxonomy profile was constructed at the above six different levels. In addition, trimmed high-quality metagenome reads were assembled using SOAPdenovo software (version 1.05) by different sizes of Kmer (51, 55, 59, 63) [22].

### Gene prediction

Using MetaGeneMark (version 2.10) to predict the assembly results of the open reading frame (ORFs) and then screened the gene sequences with length greater than 100 bp and translated them into corresponding amino acid sequences. All predicted genes (identify is greater than 95%, coverage is greater than 90%) were clustered using CD-HIT [23]. The longest sequence of genes in each category was selected to construct a non-redundant gene sets for further analyses [24].

### Functional annotation analysis

To identify the biological function and associated metabolic pathways for the differentially expressed genes, we conducted the Kyoto Encyclopedia of Genes and Genomes (KEGG) database using BLSAT (version 2.2.28+) to obtain the KEGG orthologue (KO) annotation information from KEGG database [25], and the abundance of Non-supervised Orthologous Groups (NOG) function in each sample was calculated by comparing the gene sequence with the evolutionary genealogy of genes: Non-supervised Orthologous Groups (eggNOG) database [26].

## Results

### Effect of moxibustion on DSS induced UC rats

**Fig. 2:**
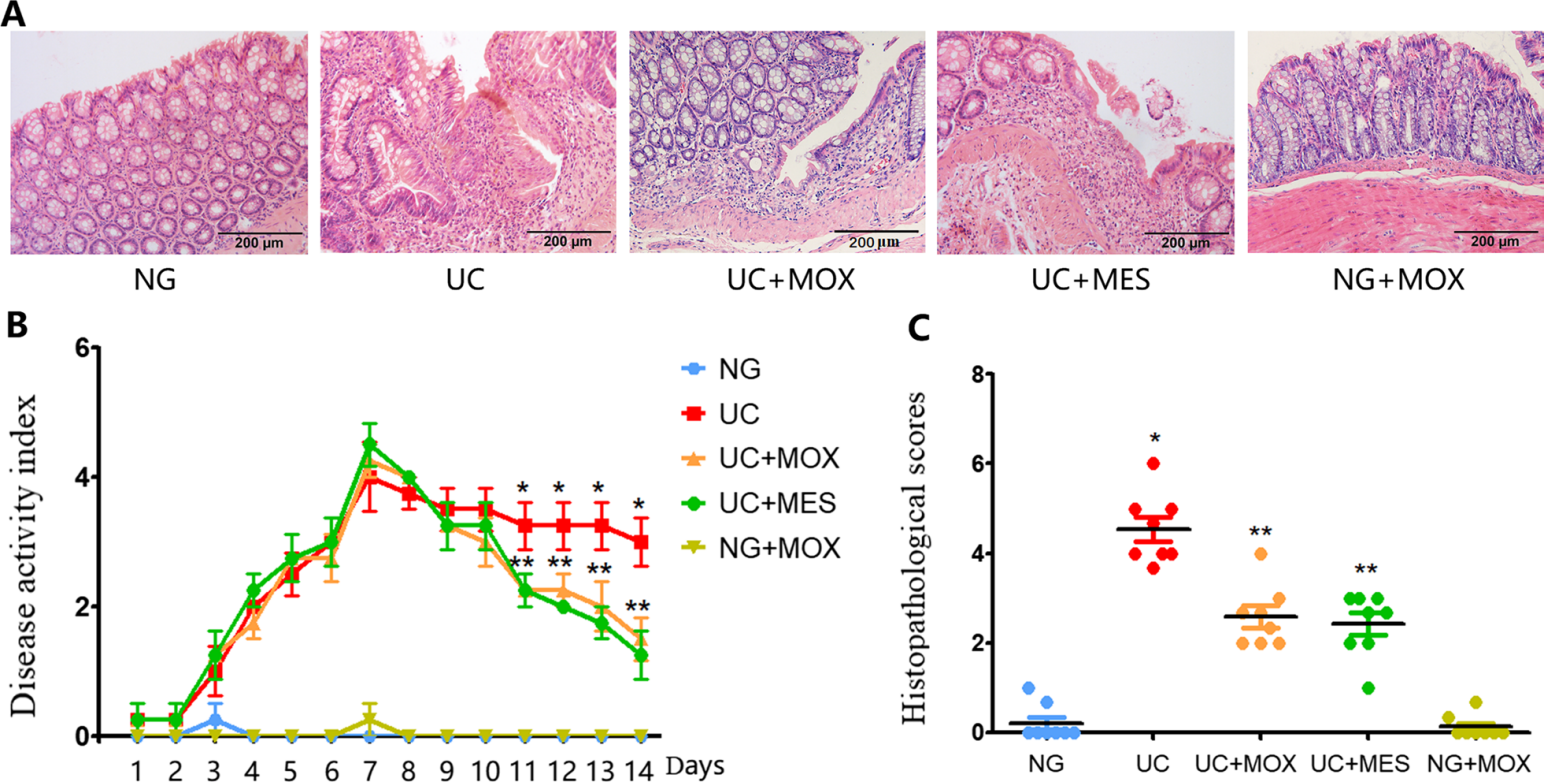
The effect of moxibustion on DSS induced UC rats. **A** Histopathological observation (HE, × 200); **B** Disease activity index; C: Histopathological scores. *P < 0.01 vs. NG group; **P < 0.01 vs. UC group. NG: normal group; UC: UC model group; UC + MOX: moxibustion group; UC + MES: mesalazine group; NG + MOX: normal rats with moxibustion group

As shown in Fig. 2A, the colonic mucosal epithelium of the NG and NG + MOX group was intact. While the mucosal epithelium of UC rats was absent, ulcer, edema could be seen with a large amount of inflammatory cells infiltrations. Compared with the UC group, histopathological observation was improved in the UC + MOX and UC + MES group, the mucosal epithelium was restored, with healed ulcer and decreased inflammatory cells infiltration. Compared with the NG group, DAI score was significantly increased in the UC group (*P* < 0.01, Fig. 2B). There was no statistically significant difference in DAI scores between the NG group and the NG + MOX group. However, rats in the UC + MOX and UC + MES groups had lower DAI scores than the UC group (*P* < 0.01). The histopathology score in the UC group was significantly increased than that in the NG group (*P* < 0.01). Conversely, the histopathology scores in the UC + MOX and UC + MES groups were significantly decreased compared to the rats in the UC group (*P* < 0.01) (Fig. 2C).

### Taxonomic characterization of faecal microbiota

**Fig. 3:**
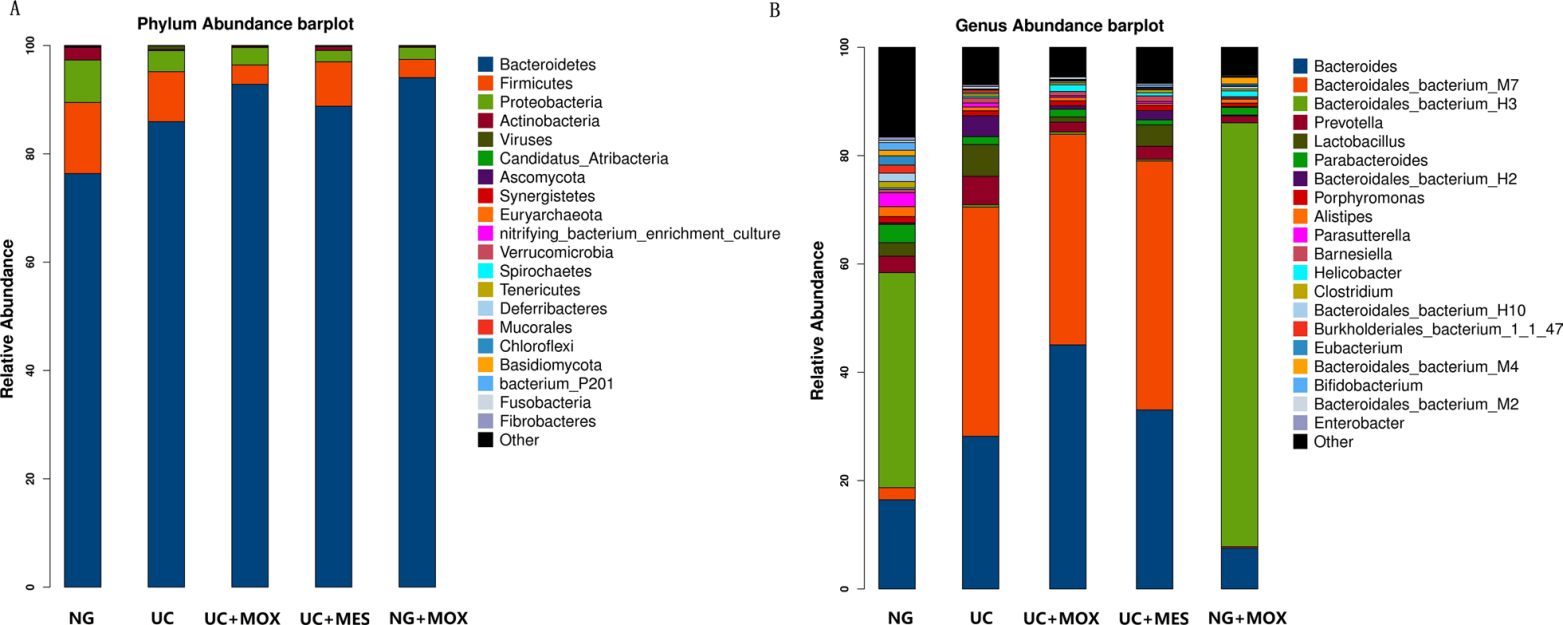
The effect of moxibustion on overall community structure at phylum and genus level. **A** at the phylum level, *Bacteroidetes* and *Firmicutes* were the most abundant taxa followed by *Proteobacteria* in five groups; **B** at the genus level, *Bacteroides*, *Bacteroides_bacterium_M7*, *Bacteroides_bacterium_H3* were the most abundant taxa followed by *Prevotella* in five groups. NG: normal group; UC: UC model group; UC + MOX: moxibustion group; UC + MES: mesalazine group; NG + MOX: normal rats with moxibustion group

We got 192,389,261,700 base pair (bp) raw bases, with an average of 25,651,902 raw reads for each sample. After quality control, we obtained an average of 23,177,046 clean reads for each. Based on the clean reads, the composition of faecal microbiota communities was revealed at the phylum and genus level. Overall, *Bacteroidetes*, *Firmicutes*, *Proteobacteria*, *Actinobacteria*, *Ascomycota*, *Synergistetes*, *Euryarchaeota*, *Verrucomicrobia*, *Spirochaetes*, *Mucorales*, *Basidiomycota* and *Fibrobacteres* were detected in all samples. Among these phyla, *Bacteroidetes*, *Firmicutes*, *Proteobacteria* and *Actinobacteria* were the four most dominant phyla, accounting for more than 98% of microorganisms in all groups. In the UC group, *Bacteroidetes* increased and *Firmicutes*, *Proteobacteria*, *Ascomycota*, *Actinobacteria*, and *Synergistetes* decreased compared with NG rats. Compared with the UC group, the UC + MOX group had an increased abundance of *Bacteroidetes*, *Actinobacteria*, *Ascomycota*, *Synergistetes* and a decreased abundance of *Firmicutes* and *Proteobacteria*. In addition, *Bacteroidetes*, *Actinobacteria*, *Ascomycota*, *Synergistetes* were increased and *Firmicutes*, *Proteobacteria* and *Deferribacteres* were decreased in the UC + MES group compared with the UC group. Compared with the NG group, the NG + MOX group had a decreased abundance of *Firmicutes*, *Proteobacteria* and *Actinobacteria* and a increased abundance of *Bacteroidetes* (Fig. 3A).

At the genus level, *Bacteroides*, *Bacteroides_bacterium_M7*, *Bacteroides_bacterium_H3* followed by the *Prevotella*, *Lactobacillus*, *Parabacteroides*, *Bacteroidales_bacterium_H2*, *Porphyromonas*, *Alistipes*, *Parasutterella*, *Barnesiella*, *Helicobacter*, *Clostridium*, *Bifidobacterium* and *Enterobacter* were detected in all groups. Compared with the NG group, the relative abundance of *Bacteroides*, *Bacteroides_bacterium_M7*, *Prevotella*, *Bacteroidales_bacterium_H2* and *Barnesiella* were increased and *Bacteroides_bacterium_H3*, *Parabacteroides*, *Porphyromonas*, *Alistipes* and *Parasutterella* reduced in the UC group. Interestingly, treatment of UC model rats with moxibustion led to a higher abundance of *Bacteroides* and *Bacteroides_bacterium_H3* and a lower of *Bacteroides_bacterium_M7*, *Prevotella*, *Bacteroidales_bacterium_H2* and *Parasutterella*. In addition, rats in the UC + MES group had a higher abundance of *Bacteroides*, *Bacteroides_bacterium_M7*, and *Barnesiella* and a lower abundance of *Bacteroides_bacterium_H3*, *Prevotella*, *Parasutterella* and *Alistipes* compared with the UC group (Fig. 3B).

**Fig. 4:**
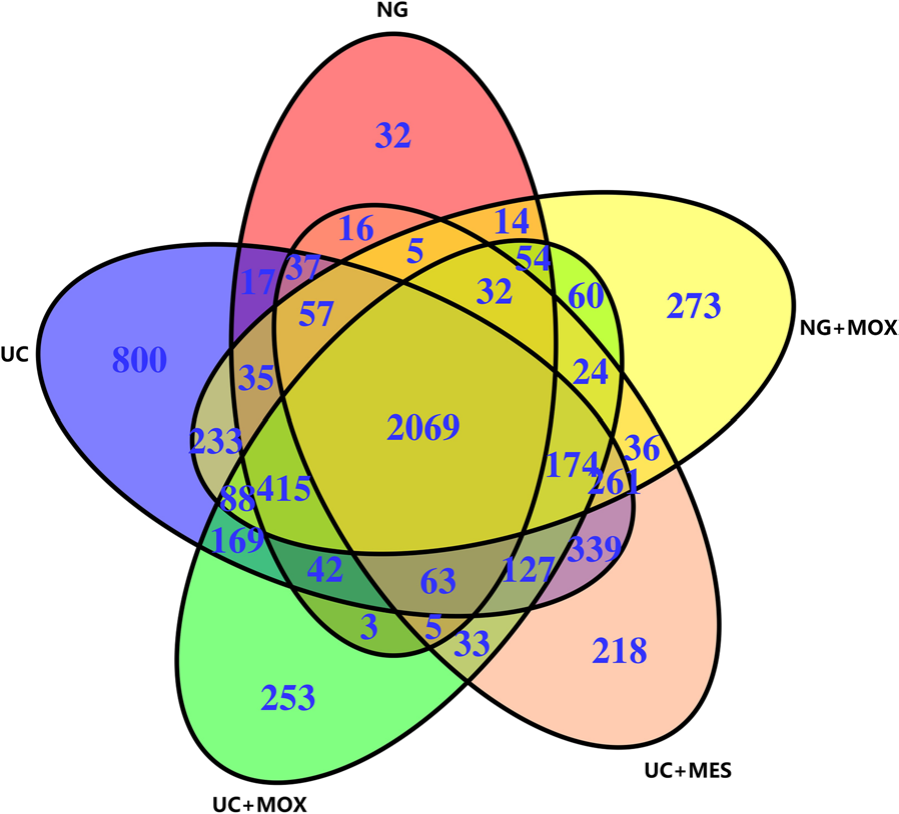
Summary of OTUs identified at the species level in all groups. There were 2069 core species were present in all groups. The UC group had the most unique species (800). NG: normal group; UC: UC model group; UC + MOX: moxibustion group; UC + MES: mesalazine group; NG + MOX: normal rats with moxibustion group

A total of 5984 species have been identified, of which 2069 are core species distributed in all groups (Fig. 4). The average number of species detected in UC group was the highest (4926), while the lowest was found in NG group (2896). A total of 3611 species were detected in the UC + MOX rats, 3496 species in the UC + MES rats and 3830 species in the NG + MOX rats. Additionally, the UC group had the most unique species (800), even after treatment with moxibustion.

**Fig. 5:**
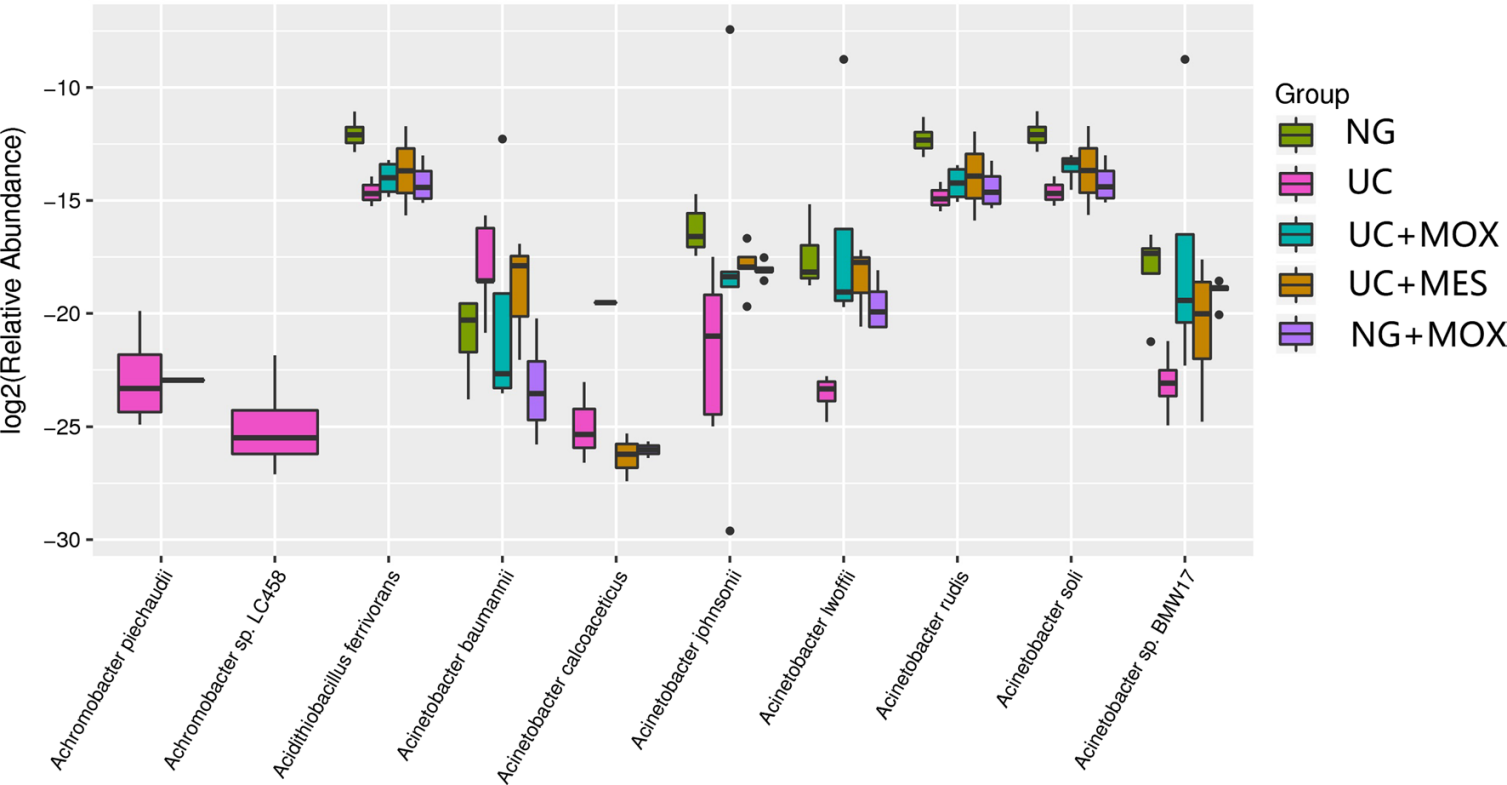
Species-level differences of gut microbiota between groups. *Achromobacter piechaudii* and *Achromobacter sp.LC458* were the absolute dominant species in the UC group. NG: normal group; UC: UC model group; UC + MOX: moxibustion group; UC + MES: mesalazine group; NG + MOX: normal rats with moxibustion group

To gain further insight into the diversity of microbial community between groups, species-level differences was analysed (Fig. 5). *Achromobacter piechaudii*, *Achromobacter sp.LC458*, *Acidithiobacillus ferrivorans*, *Acinetobacter baumannii*, *Acinetobacter calcoaceticus*, *Acinetobacter johnsonii*, *Acinetobacter lwoffii*, *Acinetobacter rudis*, *Acinetobacter soli* and *Acinetobacter sp.BMW17* were the most affected species. Additionally, *Achromobacter piechaudii* and *Achromobacter sp.LC458* were the absolute dominant species in the UC group, suggesting that it may be related to the state of UC disease. Compared with the NG group, *Achromobacter piechaudii*, *Achromobacter sp.LC458* and *Acinetobacter baumannii* were increased and *Acidithiobacillus ferrivorans*, *Acinetobacter johnsonii*, *Acinetobacter lwoffii*, *Acinetobacter rudis*, *Acinetobacter soli* and *Acinetobacter sp. BMW17* were decreased in the UC model group. In addition, we found significantly lower abundance of *Acinetobacter baumannii* in the moxibustion group than in the UC group. We also noted that *Acidithiobacillus ferrivorans*, *Acinetobacter johnsonii*, *Acinetobacter lwoffii*, *Acinetobacter rudis*, *Acinetobacter soli* and *Acinetobacter sp. BMW17* were increased in moxibustion, mesalazine and normal rats with moxibustion groups than UC group.

**Fig. 6:**
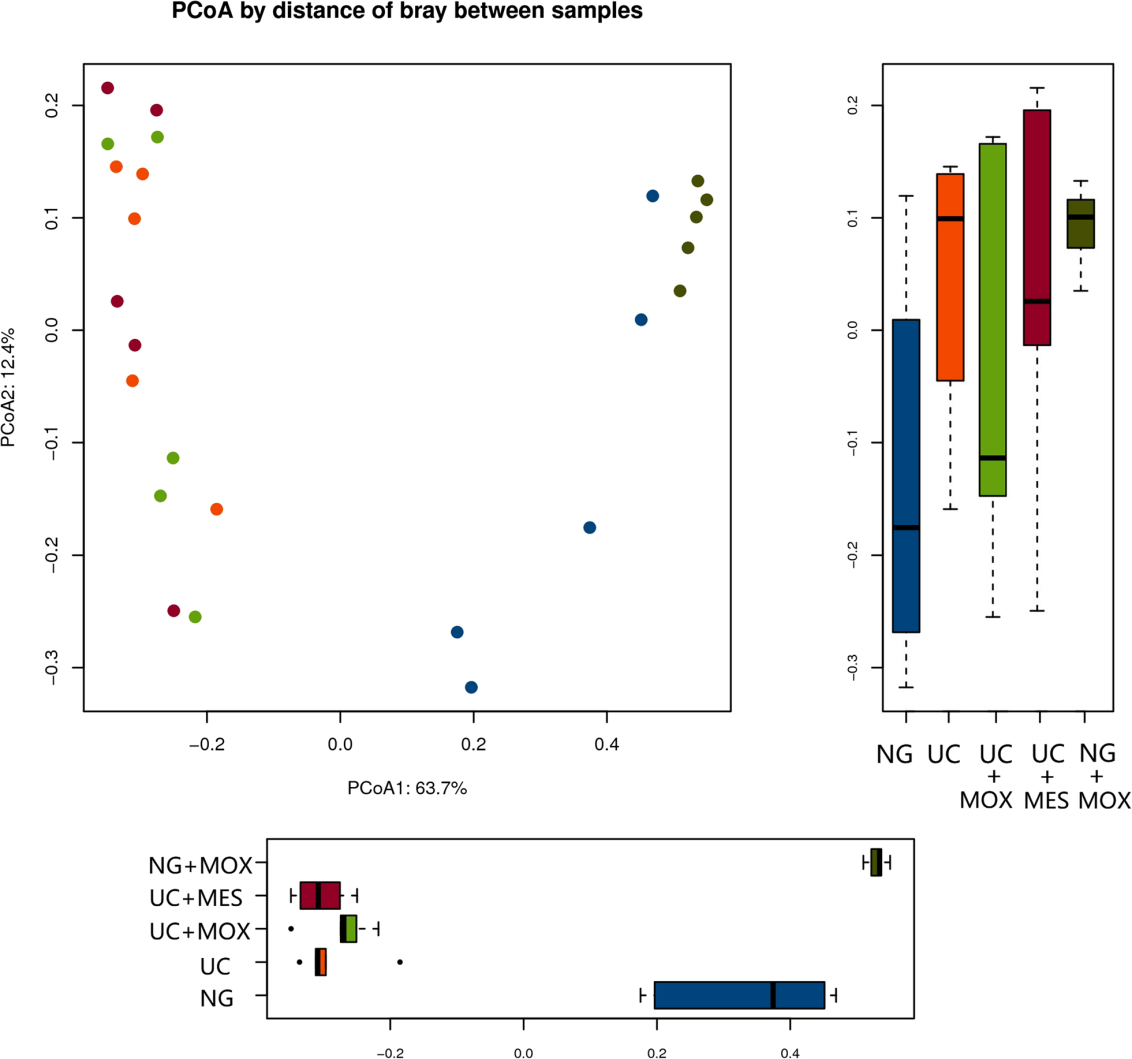
The effect of moxibustion on Beta diversity of gut microbiota. UC samples clustered separately from NG samples. The UC + MOX group was more similar to the UC + MES group on the first axis, whereas the NG + MOX group was more similar to the NG group. NG: normal group; UC: UC model group; UC + MOX: moxibustion group; UC + MES: mesalazine group; NG + MOX: normal rats with moxibustion group

A principal coordinates analysis (PCoA) was performed to compare the composition of bacterial community within 25 samples, showed a clear separation of the community composition between normal rats and UC rats (Fig. 6). PCoA by distance of bray between samples explained 63.7% of variation in the first axes. The ordinations demonstrated that the disease state was the primary factor affecting the overall differences of bacterial communities between groups. The UC + MOX group was more similar to the UC + MES group on the first axis, whereas the NG + MOX group was more similar to the NG group. It was suggested that the diversity of gut microbiota was broken in UC rats. Furtherly, the flora community composition of UC rats was changed as well.

### Correlation between the gut microbiota in species level and cytokines, LPS, SIgA in the UC model rats

**Fig. 7:**
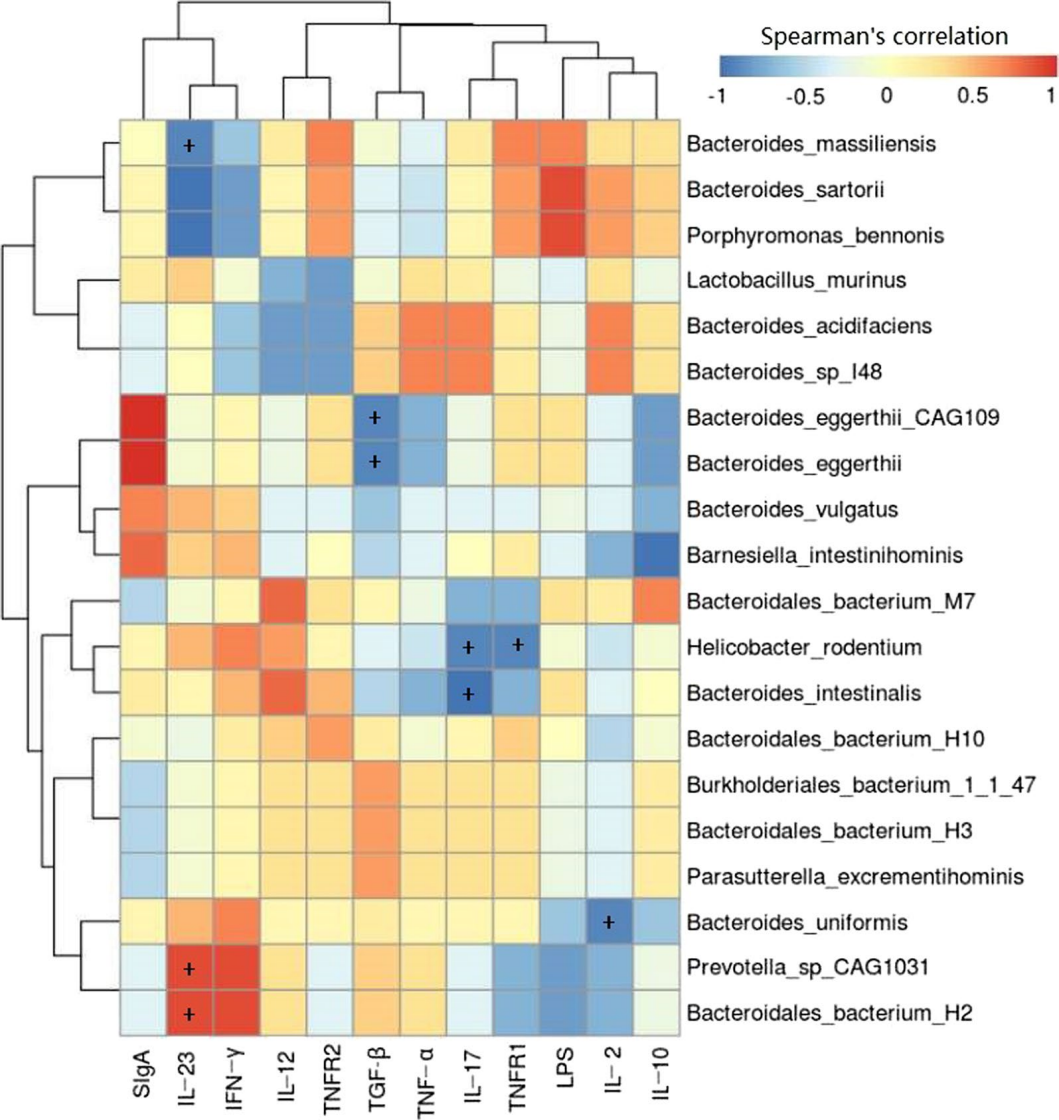
Heatmap of spearman’s correlation between the top 20 species and 12 inflammation-associated cytokines in UC group. N = 5; ^+^*P* < 0.05. The different colors represent the range of (r) from − 1 to + 1. Orange indicates a perfect positive correlation, whereas blue indicates a perfect negative correlation

Spearman correlation analysis between the gut microbiota and cytokines (IL-2, IL-10, IL-12, IL-17, IL-23, TGF-β, IFN-γ, TNF-α, TNFR1 and TNFR2), LPS, SIgA in serum in the UC model rats listed in Fig. 7. Our previous studies showed that compared with the UC rats, the levels of IL-6, IL-12, IL-17, IL-23, IFN-γ, LPS, TNF-α, TNFR1, TNFR2 in the moxibustion group were decreased and IL-2, TGF-β were increased [20]. From the figure, we found that *Bacteroides_massiliensis* was negatively (*P* < 0.05) correlated with IL-23, *Bacteroides_eggerthii_CAG109* and *Bacteroides_eggerthii* were negatively (*P* < 0.05) correlated with TGF-β. A significant negative (*P* < 0.05) correlation also can be found between *Helicobacter_rodentium* and *Bacteroides_intestinalis* and IL-17. *Helicobacter_rodentium* was also negatively (*P* < 0.05) correlated with TNFR1, *Bacteroides_uniformis* was negatively (*P* < 0.05) correlated with IL-2. Additionally, IL-23 exhibited significant positive (*P* < 0.05) correlation with the species *Prevotella_sp_CAG1031* and *Bacteroides_bacterium_H2*.

### Functional annotation analysis

**Fig. 8:**
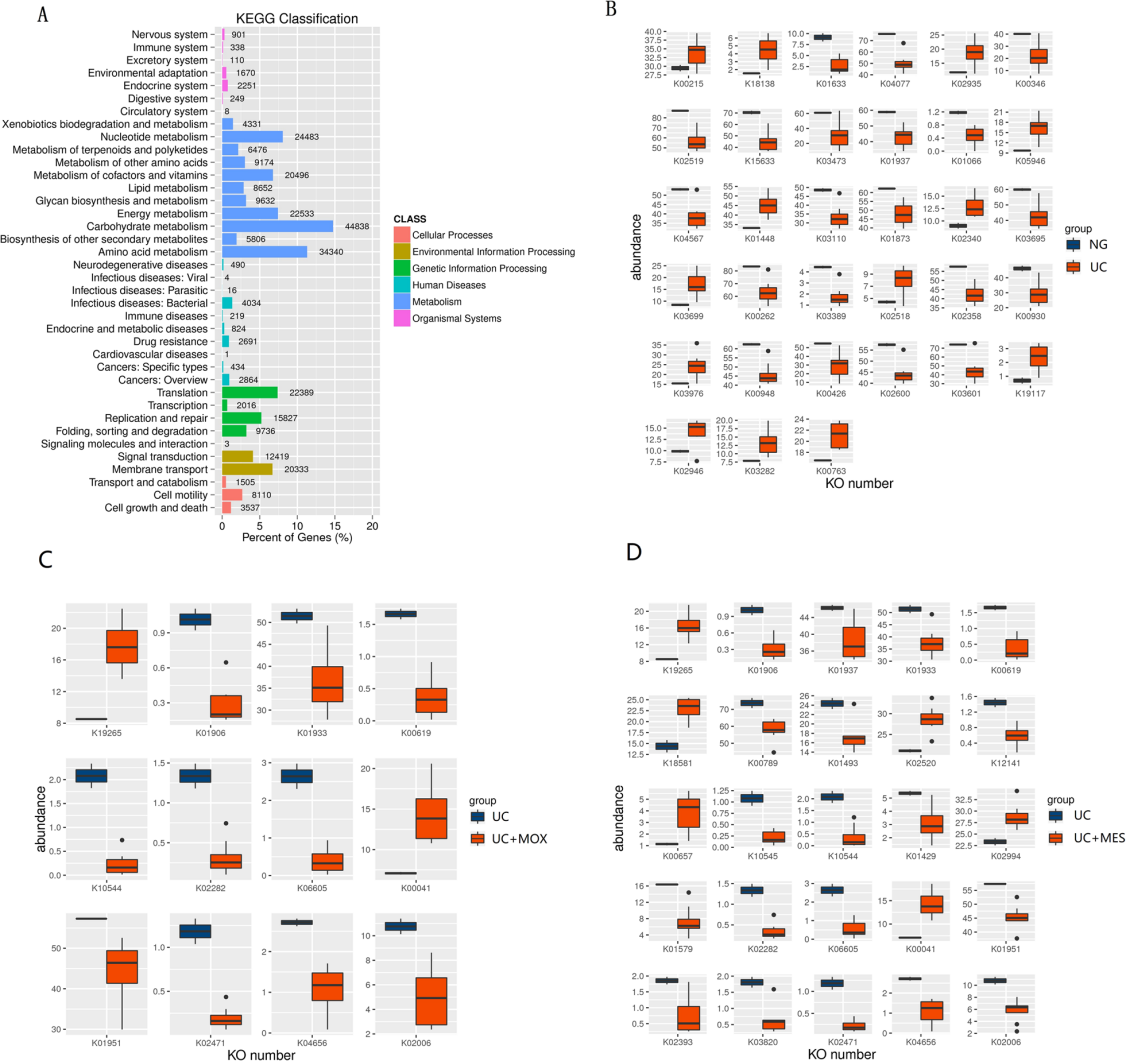
Classification of KEGG annotations of gut microbiota in all samples. **A** Summary of KEGG classification across all samples. **B** Differential KEGG orthologies (KOs) between NG and UC groups. **C** Differential KEGG orthologies (KOs) between UC and UC + MOX groups. **D** Differential KEGG orthologies (KOs) between UC and UC + MES groups. NG: normal group; UC: UC model group; UC + MOX: moxibustion group; UC + MES: mesalazine group; NG + MOX: normal rats with moxibustion group

We used metagenomics methodologies to understand the functional capacity potential of gut microbiome in the UC progression. KEGG orthologs (KOs) is a taxonomic system of proteins (enzymes) that are highly similar in sequence. In total, 30,3740 KEGG genes were found and assigned to 38 KEGG pathways. The five functional modules were metabolism (62.81%), genetic information processing (16.45%), environment information processing (10.78%), cellular processes (4.33%), human diseases (3.81%) and organismal systems (1.82%). In five metabolic pathways, the most predominant functional categories were carbohydrate metabolism (44,838 genes, 14.76%), amino acid metabolism (34,340 genes, 11.31%), nucleotide metabolism (24,483 genes, 8.06%), energy metabolism (22,533 genes, 7.42%), translation (22,389 genes, 7.37%), membrane transport (20,333 genes, 6.69%) and replication and repair (15,827 genes, 5.21%) (Fig. 8A). Then we analyzed the differential KOs between NG and UC rats, and identified 33 KOs showing different abundance. Twenty out of these 33 KOs had the higher abundance in the NG rats, including those KOs associated with metabolism of cofactors and vitamins (K01633 and K03473), transport and catabolism (K04077), and carbohydrate metabolism (K15633). The other 13 KOs were enriched in UC rats, such as amino acid metabolism (K00215), translation (K02935), signaling molecules and interaction (K03699) (Fig. 8B). We also analyzed the differential KOs between UC and UC + MOX rats, and identified 12 KOs showing different abundance. Ten out of these 12 KOs had the higher abundance in the UC rats, including those KOs associated with nucleotide metabolism (K01933 and K01951) and membrane transport (K02471). The other 2 KOs were enriched in UC + MOX rats associated with Carbohydrate metabolism (K19265 and K00041) (Fig. 8C). Similarly, we found that there was 25 KOs showing different abundance between UC and UC + MES rats. Nineteen out of these 25 KOs had the higher abundance in the UC rats, including those KOs associated with metabolism of cofactors and vitamins (K01906), nucleotide metabolism (K01937, K01933, K01493, K01951 and K01429) and membrane transport (K02471, K10544, K02282 and K02006). The other 6 KOs were enriched in UC + MES rats associated with Carbohydrate metabolism (K19265 and K00041) and Amino acid metabolism (K00657) (Fig. 8D).

**Fig. 9:**
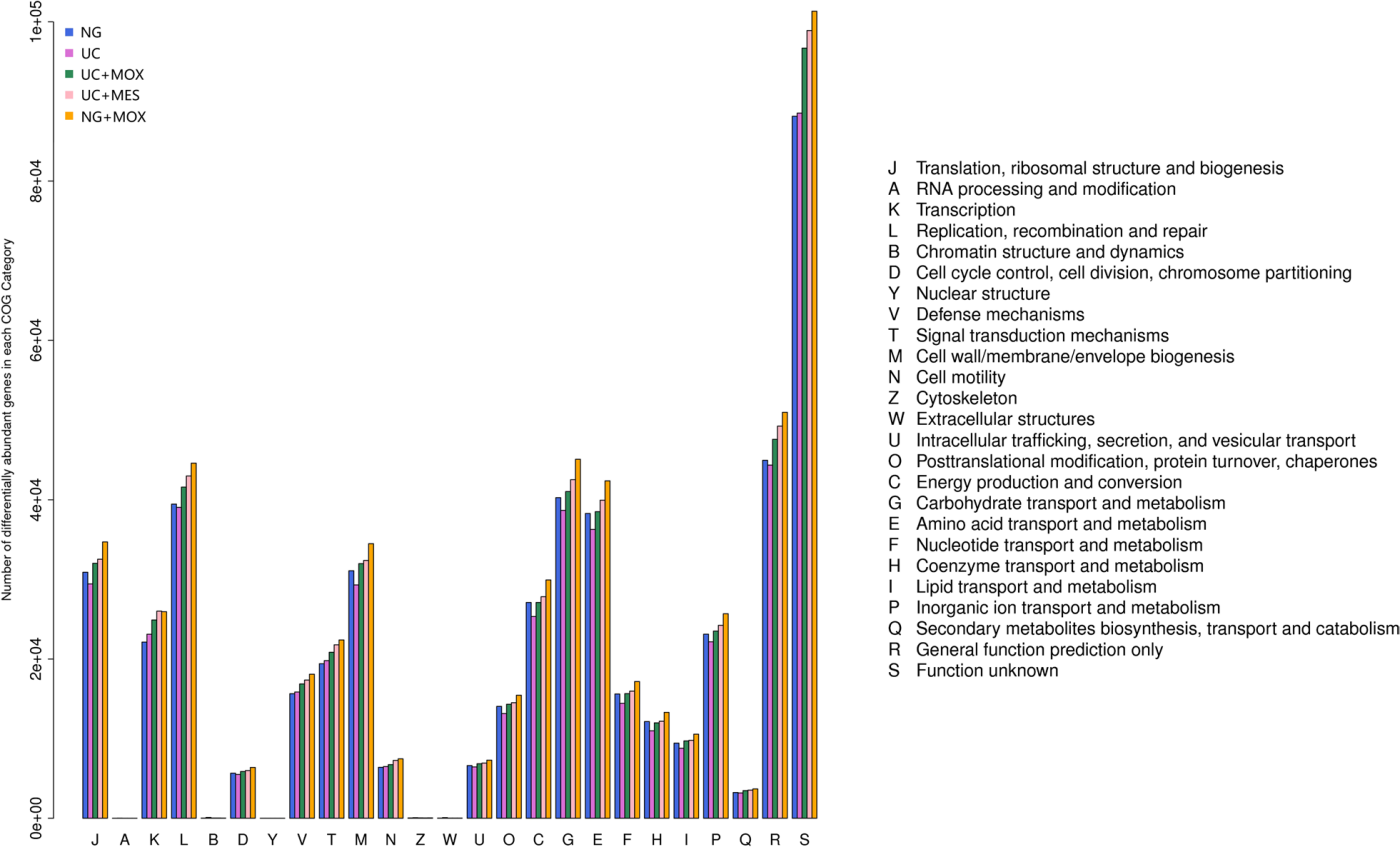
Classification of eggNOG annotations of gut microbiota in all samples. The capital letters on the X-axis represent different eggNOG categories. Y-axis shows the number of unigenes in each eggNOG category. NG: normal group; UC: UC model group; UC + MOX: moxibustion group; UC + MES: mesalazine group; NG + MOX: normal rats with moxibustion group

The EggNOG database is another common method for functional annotation using the Smith-Waterman alignment algorithm. We found that all genes were classified into 25 categories. Among these 25 functional classes, genes involved in certain metabolic pathways, such as energy production and conversion(25,339 and 27,102, respectively), amino acid transport and metabolism (36,275 and 38,286, respectively), nucleotide transport and metabolism (14,437 and 15,610, respectively), carbohydrate transport and metabolism (38,675 and 40,273, respectively), replication, recombination and repair (39,060 and 39,466, respectively), were under-represented in UC model rats compared with NG rats. It was suggested that the less vigorous microbial growth and metabolic stage in UC rats compared with that of normal rats. In addition, these changes in the metabolic pathways could be reversed by moxibustion treatment and mesalazine treatment (Fig. 9).

### KEGG annotation exposes metabolic differences between UC and moxibustion treatment rats

**Fig. 10:**
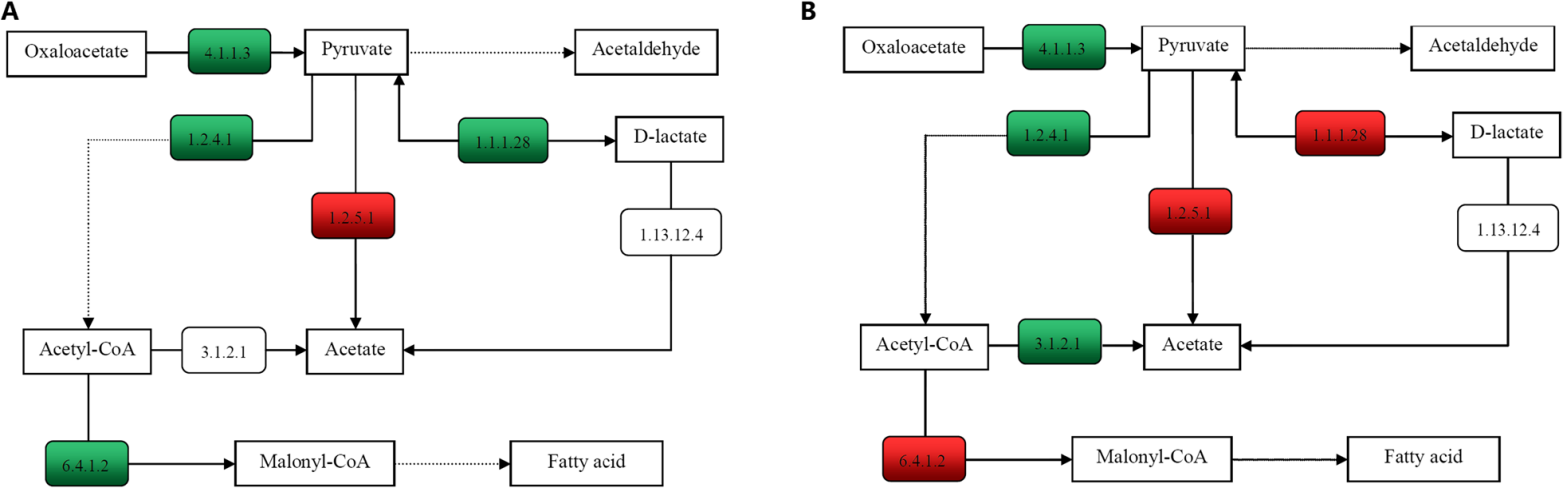
Functional variations in pyruvate metabolism. **A** between NG and UC groups, the numbers in the red box represent genes in the UC group that were more abundant than in the normal group, whereas the green box represent genes that were less abundant in the UC group. **B** between UC and UC + MOX groups, the numbers in the red box represent genes in the UC + MOX group that were more abundant than in the UC group, whereas the green box represent genes that were less abundant in the UC + MOX group. NG: normal group; UC: UC model group; UC + MOX: moxibustion group

Marker genes involved in key processes of pyruvate metabolism that were significantly enriched in NG, UC or UC + MOX rats were annotated on KEGG pathway (Fig. 10). Oxaloacetate, which is an important intermediate in the tricarboxylic acid cycle, is used to produce pyruvate by oxaloacetate decarboxylase (EC 4.1.1.3). Compared with the NG group, pyruvate-producing genes (ECs 4.1.1.3 and 1.1.1.28) were reduced in the UC group. Compared with the UC group, pyruvate-producing genes (EC 1.1.1.28) was increased in the UC + MOX group. In the UC group, EC 1.2.4.1 involved in the reduction of pyruvate to acetyl-CoA which is a central metabolite of energy metabolism was also reduced compared with the NG group. EC 6.4.1.2 (Acetyl-CoA carboxylase), reduced in the UC group, was responsible for Acetyl-CoA to become malonyl-CoA which plays an important role in the biological synthesis of fatty acids and the biosynthesis of polyketones. Compared with the UC group, EC 6.4.1.2 (Acetyl-CoA carboxylase), increased in the UC + MOX group. In summary, the metabolism of fatty acids in the UC group was decreased compared with the NG group, and increased in UC + MOX group to some extent compared with the UC group.

## Discussion

Our body harbours enormous quantities of microorganisms that reside in the gut, skin, nasal, oral cavities and vagina [27]. The microbiota colonized in the gut is one of the most important habitats and can regulates the intestinal mucosal barriers, promotes energy intake, absorption and storage, mediates development of the host immune system, and prevents propagation of pathogenic microorganisms [28–31]. Previous studies have shown that dysbiosis of the gut microbiota can cause a large spectrum of human diseases, such as inflammatory bowel disease, colorectal cancer, obesity, type 1diabetes [8, 32–34]. In this study, our data showed that there were some differences between normal and ulcerative colitis rats in the gut microbiota, which is consistent with the previous study. Previous studies have shown that moxibustion is a safe and effective treatment for UC [15, 35–37], but its specific mechanism has not yet been fully understood. Therefore, we used metagenomic sequencing to determine the effects of moxibustion treatment on the microbial compositions and functional variations in a UC rat model.

**Fig. 11:**
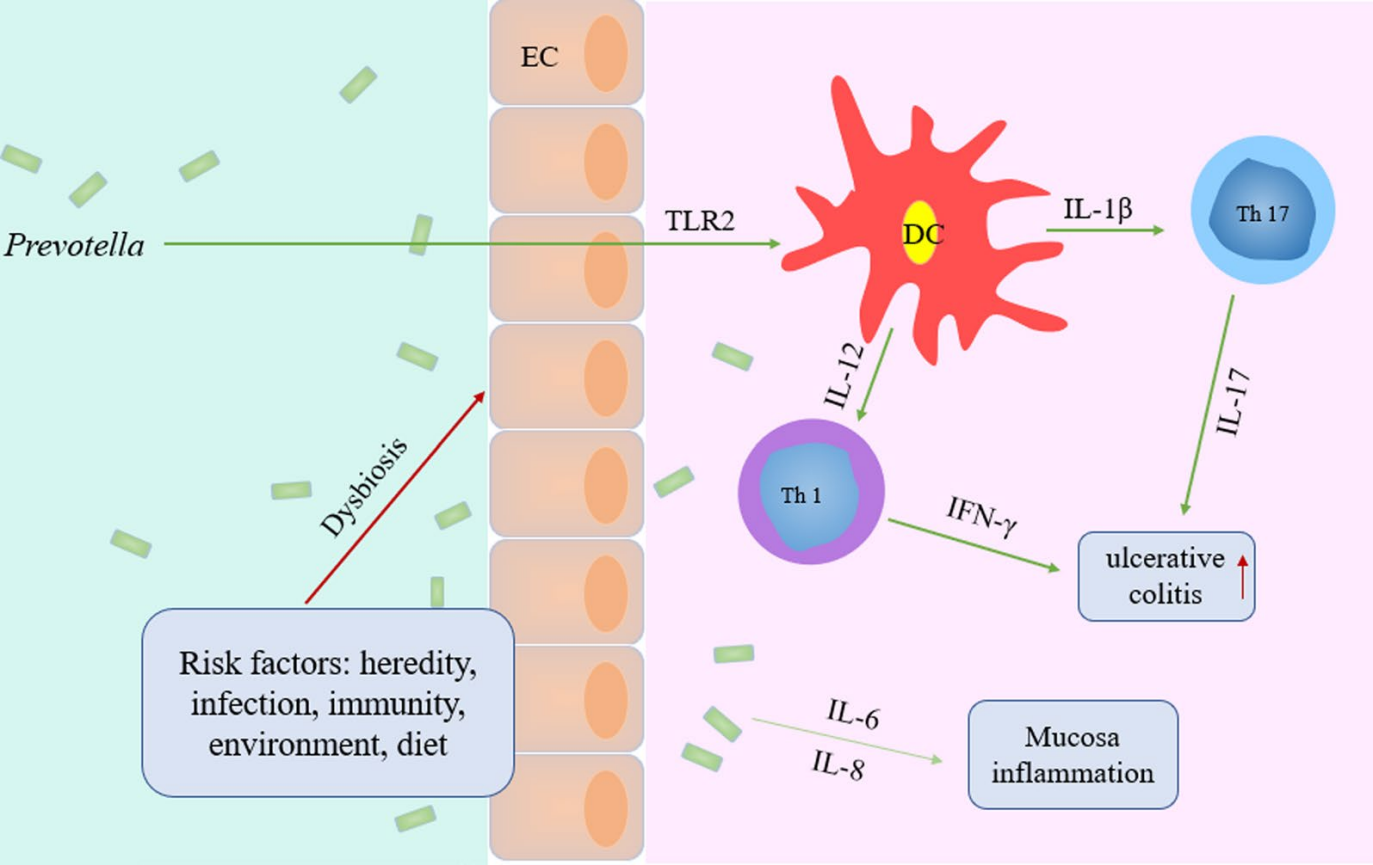
*Prevotella*‐mediated inflammation in DSS colitis. *Prevotella* stimulate the release of IL‐1β and IL-12 by dendritic cells (DC) through TLR2, which in turn mediates IL‐17 and IFN-γ production by Th17 and Th1 cells. Dysbiosis‐associated increase in Th17 and Th1 immune responses may affect ulcerative colitis

We found that *Bacteroidetes* and *Firmicutes* were the dominant taxa at phylum level, followed by *Proteobacteria* in all groups, it was consistent with our previous study using 16S rRNA amplicon sequencing [20]. Previous studies have found many bacteria associated with UC, and some of our findings are consistent with previous studies. Studies reported that *Prevotella* predominantly activate Toll‐like receptor 2, leading to production of T helper type 17 (Th17) polarizing cytokines and Th1 by dendritic cells, including IL‐23, IL‐1 and IFN-γ [38]. Furthermore, *Prevotella* has been identified to be linked with elevated IL-6, IL-8, IL-17 and CCL20 producing cells in the mucosa [38, 39] and enriched in IBD patients [40] (Fig. 11). Consistent with the studies, we found that the relative abundance of *Prevotella* was increased in UC rats compared with HC rats, indicating an association with ulcerative colitis. It was indicated that *Prevotella* may be an important pathological organism involved in ulcerative colitis by promoting chronic inflammation. In our study, the relative abundance of *Parabacteroides* was decreased in UC rats compared with HC rats. Studies have found that *Parabacteroides* can increase the level of gamma-aminobutyric acid (GABA) in the brain [41]. GABA is an important nerve signal substance in the mature central nervous system (CNS). Emerging evidence indicates that GABA has many beneficial effects such as ameliorating immune and inflammatory response. GABA inhibits pro-Inflammatory cytokine such as IL-12, IFN-γ, and TNF-α production, while significantly increasing tissue levels of IL-10 in DSS-induced colitis [42]. Therefore, the abnormality of the relative abundance of *Parabacteroides* may be the key to the pathogenesis of UC, this appears to be a novel finding that will require additional research.

Notably, we detected a significant higher abundance of *Acinetobacter baumannii* in UC rats. *Acinetobacter baumannii*, a gram-negative bacterium, is an opportunistic pathogen capable of causing a variety of infections (pulmonary, wound, skin, urinary tract, and bloodstream infections) in susceptible hosts [43, 44]. At the species level, the *Achromobacter piechaudii* and *Achromobacter sp.LC458* were the absolute dominant species in the UC group and barely detectable in other groups. In previous studies, researchers have reported that *Achromobacter piechaudii* can act as an opportunistic pathogen cause certain human diseases [45, 46]. Accordingly, we can infer that those two bacterium may be related to the state of UC disease and warrant further investigation. Compared with normal rats, the abundance of some species such as Acidithiobacillus ferrivorans, Acinetobacter johnsonii were decreased in UC rats. In addition, the relative abundance levels of several opportunistic pathogenic species were increased in the UC rats. Thus, there was a significant difference in community composition between normal rats and UC rats, which was confirmed by PCoA.

Interestingly, we found that moxibustion and mesalazine treatment decreased the abundance levels of *Prevotella*. *Prevotella*‐mediated mucosal inflammation leads to accumulation of intestinal inflammatory mediators and bacterial products, which in turn may affect the occurrence and development of ulcerative colitis. Thus, we have reason to believe that moxibustion could adjust the inflammatory response of ulcerative colitis by the changes of the intestinal microbiota community. We also found that moxibustion treatment increased the abundance levels of *Bacteroides* and decreased *Prevotella* abundance. *Bacteroides* has been found to induce CD4 + T cells by enhancing anti-inflammatory IL-10 and suppressing pro-inflammatory IL-17 production through secreting polysaccharide A which is not only able to prevent but also cure experimental colitis in animals [47, 48]. *Prevotella* can stimulate inflammatory cytokines, such as IL-17 [38], *Bacteroides* can up-regulate anti-inflammatory IL-10 and down-regulate inflammatory cytokines IL-17. Our previous studies showed that compared with the UC rats, the levels of IL-6, IL-12, IL-17, IL-23, IFN-γ, LPS, TNF-α, TNFR1, TNFR2 in the moxibustion group were decreased and IL-2, TGF-β were increased [20]. It suggested that moxibustion treatment may protect the host from mucosal inflammation by modulating the intestinal microbiota community (such as *Prevotella* and *Bacteroides)* to down-regulating inflammatory cytokines and up-regulating anti-inflammatory cytokines. In addition, spearman correlation analysis between the intestinal flora and cytokines such as IL-6, IL-12 indicated that the intestinal flora is closely related to the secretion and expression of cytokines in ulcerative colitis, and they interacted with each other to regulate immune function in the body. Moxibustion can adjust the balance of intestinal microecology and restore the immune system, so as to ameliorate DSS-induced ulcerative colitis. Moxibustion can regulate the intestinal flora through multi-faceted and multi-level synergistic effects, but the specific mechanism is still unclear, and further research is needed. At present, the research on the effect of moxibustion on the intestinal flora is mainly focuses on regulating the intestinal microbiota by regulating brain-gut peptides and inflammatory response [49]. In this study, we found that moxibustion can promote the repair of mucous membrane in UC rats and improve the microbiome. However, the underlying mechanisms may require further study through animal and cell experiments. In the future, we can pay more attention to the warming effect of moxibustion, acupoint specificity, acupoint combination and different intervention measures to better study the specific mechanism of moxibustion in regulating intestinal flora.

## Conclusions

In conclusion, based on the analysis of the Illumina Hiseq platform, moxibustion improved the richness and diversity of microbial community in rats with ulcerative colitis, and after moxibustion, the composition and diversity of the intestinal flora of the UC group were restored. Opportunistic pathogens decreased (such as *Prevotella*), while the abundance of *Bacteroides* was elevated in various degrees. Thus, moxibustion treatment could protect the host from mucosal and ameliorate intestinal inflammation of DSS-induced ulcerative colitis by modulating the intestinal microbiota community.

## Data Availability

The datasets generated and/or analysed during the current study are available in the NCBI repository, accession: PRJNA795867.

## Abbreviations

UC: Ulcerative colitis
DSS: Dextran sulphate sodium
ORFs: Open reading frame
KEGG: Kyoto Encyclopedia of Genes and Genomes
NOG: Non-supervised Orthologous Groups
PCoA: Principal coordinates analysis
